# Initial Results Obtained with the First TWIN VLBI Radio Telescope at the Geodetic Observatory Wettzell

**DOI:** 10.3390/s150818767

**Published:** 2015-07-30

**Authors:** Torben Schüler, Gerhard Kronschnabl, Christian Plötz, Alexander Neidhardt, Alessandra Bertarini, Simone Bernhart, Laura la Porta, Sebastian Halsig, Axel Nothnagel

**Affiliations:** 1 Geodetic Observatory Wettzell, Federal Agency for Cartography and Geodesy, Sackenrieder Str. 25, 93444 Bad Koetzting, Germany; E-Mails: gerhard.kronschnabl@bkg.bund.de (G.K.); christian.ploetz@bkg.bund.de (C.P.); 2 Faculty of Aerospace Engineering, University of the Federal Armed Forces Munich, 85777 Neubiberg, Germany; 3 Geodetic Observatory Wettzell, Technische Universität München, Sackenrieder Str. 25, 93444 Bad Koetzting, Germany; E-Mail: neidhardt@fs.wettzell.de; 4 Institute of Geodesy and Geoinformation, University of Bonn, Nußallee 17, 53115 Bonn, Germany; E-Mails: abertari@mpifr-bonn.mpg.de (A.B.); simone@mpifr-bonn.mpg.de (S.B.); laporta@mpifr-bonn.mpg.de (L.P.); sebastian.halsig@uni-bonn.de (S.H.); nothnagel@uni-bonn.de (A.N.); 5 Max Planck Institute for Radio Astronomy, Auf dem Hügel 69, 53121 Bonn, Germany

**Keywords:** Very Long Baseline Interferometry (VLBI), VLBI Global Observing System (VGOS), Geodetic Observatory Wettzell, TWIN Telescopes Wettzell, VLBI correlation

## Abstract

Geodetic Very Long Baseline Interferometry (VLBI) uses radio telescopes as sensor networks to determine Earth orientation parameters and baseline vectors between the telescopes. The TWIN Telescope Wettzell 1 (TTW1), the first of the new 13.2 m diameter telescope pair at the Geodetic Observatory Wettzell, Germany, is currently in its commissioning phase. The technology behind this radio telescope including the receiving system and the tri-band feed horn is depicted. Since VLBI telescopes must operate at least in pairs, the existing 20 m diameter Radio Telescope Wettzell (RTW) is used together with TTW1 for practical tests. In addition, selected long baseline setups are investigated. Correlation results portraying the data quality achieved during first initial experiments are discussed. Finally, the local 123 m baseline between the old RTW telescope and the new TTW1 is analyzed and compared with an existing high-precision local survey. Our initial results are very satisfactory for X-band group delays featuring a 3D distance agreement between VLBI data analysis and local ties of 1 to 2 mm in the majority of the experiments. However, S-band data, which suffer much from local radio interference due to WiFi and mobile communications, are about 10 times less precise than X-band data and require further analysis, but evidence is provided that S-band data are well-usable over long baselines where local radio interference patterns decorrelate.

## 1. Introduction

Geodetic VLBI telescopes observe radio sources at frequencies around 2 GHz (S-band) and 8 GHz (X-band). Signals from these distant quasi-stellar objects (Quasars) are received and recorded by at least two telescopes simultaneously. These signals are transferred to a correlator facility and cross-correlated. The geometrical delay between the signal arrival time at the two telescopes, as derived from the correlation process, is a measure of the baseline length [1,2]. Geodetic VLBI has become an important space geodetic technique, because it is the only state-of-the-art technique to establish a precision link between the inertial and the Earth-fixed reference frame. In addition, it is the only technique to determine the difference between the Coordinated Universal Time UTC, an atomic timescale, and UT1, the principal form of the Universal Time, which is tied to the rotation of the Earth [3,4].

### 1.1. VGOS—Modernization of Geodetic VLBI

Increasing requirements such as accessibility to the global reference frame at a level of 1 mm, as required for unambiguous monitoring of sea level rise for instance, can hardly be met with traditional geodetic VLBI telescopes. The next generation VLBI technology and network, the VLBI Global Observing System (VGOS), was designed and published [5], corresponding requirements were set up [6] keeping existing challenges in mind [7].

One important aspect are high slew-speed antennas (around 12°/s in azimuth an 6°/s in elevation) that can collect observations from many more radio sources delivering an increased number of data samples and thus increased precision. Increasing antenna speed reduces source switching time and facilitates the application of small-aperture antennas (typical diameters of systems currently under realization are between 12 and 13.2 m). These antennas have to fulfill strong geometric stability requirements for geodetic purposes. Typical antenna apertures of current geodetic VLBI telescopes are between 20 and 40 m. Since aperture decreases in favor of increasing speed, antenna sensitivity will—in principle—also drop down. This drawback is compensated by the utilization of state-of-the-art components, including a high degree of digital technology, as well as the use of broadband receiving systems which can capture signals in bands of up to 1 GHz.

### 1.2. TWIN Telescopes of the Geodetic Observatory Wettzell

The TWIN radio telescope pair at the Geodetic Observatory Wettzell, Germany, was officially inaugurated in May 2013 after completion of the mechanical construction. Although both antennas have the same appearance, their interior differs in terms of the receiving system. TWIN Telescope Wettzell 1 (TTW1), the first of the two new 13.2 m diameter telescopes, was finalized between June 2013 and May 2014 and entered its commissioning phase in June 2014. Although the design is closely aligned to the VGOS specifications [6] with a diameter slightly exceeding the corresponding requirement of 10–12 m, the main difference is the receiving system. In contrast to the second TWIN telescope, TTW2, which will feature a broadband feed horn from 2 to 14 GHz, TTW1 is equipped with a high-performance triple band feed horn featuring S-, X- and Ka-band frequency ranges in dual circular polarization mode. The main reason is to explore the possibilities for Ka-band geodetic VLBI up to 32 GHz, which has not been investigated in detail so far, and is also not a dedicated topic of this paper. However, it should be noted that X-band receiving capabilities of TTW1 are particularly broad ranging from approximately 6.8 up to 9.8 GHz making it usable for both legacy operations together with existing S-/X-band radio telescopes such as the 20 m Radio Telescope Wettzell (RTW) at Wettzell and future broad band telescopes such as TTW2. Section 2 of this paper will describe the technology behind TTW1 in comparison to the RTW telescope.

### 1.3. TTW1 Performance Evaluation

The initial performance evaluation is carried out at the local level. VLBI is a relative technique, *i.e.*, two telescopes must sample identical radio sources synchronously. Such a local baseline is available using the 20 m RTW together with the 13.2 m TTW1. Of course, the use of RTW will limit usable frequencies and subsequent correlation and data analysis to the traditional S- and X-band frequencies in single-polarization mode. Nevertheless, an initial—albeit not fully comprehensive—performance evaluation is possible and suitable for serving as a first end-to-end test of the system. Correlation results will be presented in Section 3 of this paper.

The advantage of using the short baseline of 123 m between RTW and TTW1 is that disturbing effects complicating very long baseline processing are almost not present here, except for local radio frequency interference. This is particularly true for tropospheric propagation effects. In fact, atmospheric refraction is still considered as one of the major challenges in microwave techniques such as VLBI [7], but the delay caused by refraction is very similar for closely spaced antennas, and hence cancels out in the correlation process. The drawback of local baseline analysis is that radio frequency interference will leave systematic patterns in the signal which are spatially correlated between RTW and TTW1 due to the short distance. Our experience with TTW1 is that the ring focal design of the new antenna, designed for particular broadband receiving capabilities, leads to an increased affliction to radio frequency interference, which is also present in the RTW, but less expressed since the passband is limited to just 900 MHz.

Consequently, as portrayed in Section 4, the local baseline between RTW and TTW1 can be determined with an accuracy of 1 to 2 mm (3D distance) using X-band data, but only about 10 times less precise in S-band. The frequency window that can be used in S-band in combination with the RTW telescope is naturally smaller compared to X-band. This leads to a decreased precision of the group delay, but does not fully explain the precision decrease actually observed.

### 1.4. Paper Summary

The following paper is divided into three main sections, starting with the technical description of the TWIN telescope TTW1. The basic characteristics of this ring focal antenna are given. The main design criterions are discussed, and the receiving system consisting of a tri-band feedhorn covering frequencies in S-, X- and Ka-band is depicted. Both the legacy S/X down-converter, used in 24 out of 26 experiments presented in this paper, and the final tri-band down-converter are illustrated followed by the description of the backend. A detailed setup giving the channel bandwidths and sample rates for the two different types of experiments analyzed later in Section 4 is provided.

Section 3 provides a summary with respect to VLBI baseline correlation featuring both correlation results for the short baseline between the 20 m telescope RTW and the new TTW1 as well as a long baseline correlation sample between TTW1 and Tsukuba. The problem of radio frequency interference (RFI) in S-band, in particular at local level, is addressed and also graphically illustrated.

Finally, Section 4 provides the initial processing results. Most of the 26 experiments analyzed are “Intensives” (duration of 1 h). In addition, results of three experiments with a duration of 24 h are presented. The digital backend in use is a DBBC-2. Moreover, six experiments were additionally sampled with an ADS3000+ backend in parallel. Data analysis of the short baseline RTW-TTW1 data is carried out for S- and X-band separately, clearly revealing that RFI-problems in S-band do exist. The 3D-difference between the X-band results and the local terrestrial survey is less than 2 mm or equal to that value in 65% of the initial 24 experiments using the S/X down-converter. Long baseline processing is also performed to resolve the short baseline indirectly using S-band data for ionospheric delay reduction of the X-band group delays. These results show deviations from the surveyed 3D-distance between 0.1 and 0.9 mm. As also indicated in the discussion of the correlation results in Section 3, this confirms that useful results can be obtained over long baselines using S-band data despite of the RFI-problems present.

## 2. TTW1 Technical Aspects

RTW, the first VLBI radio telescope at the Geodetic Observatory Wettzell, started operation in 1983. It was the first VLBI system in Germany exclusively dedicated to geodetic purposes and is frequently in use for experiments within the International VLBI Service for Geodesy and Astrometry (IVS [8]). Around 20 years later, the planning and realization of two new TWIN telescopes was initiated.

### 2.1. Specifications and Basic Characteristics (Common to TTW1 and TTW2)

**Figure 1:**
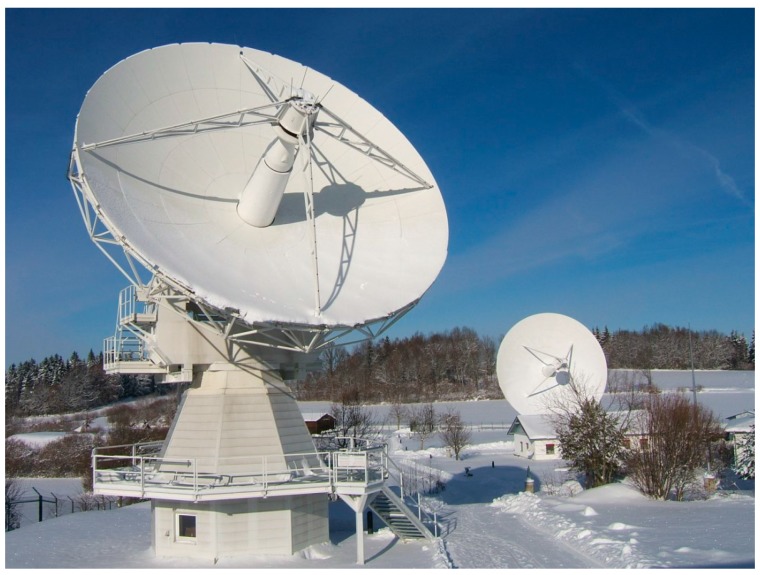
Photograph of the new 13.2 m diameter TTW1 radio telescope (left, foreground) and the 20 m diameter RTW (right, background) at the Geodetic Observatory Wettzell, Germany.

The TWIN project comprises two fast slewing radio telescopes. Both telescopes are designed for continuous 24/7 operation (entire week, entire day). The TTW antennas follow a different reflector concept compared to the RTW, see Figure 1: the ring focal design [9,10,11]. This antenna system unifies both, the advantages of a dual offset antenna, such as a high potential to reach low ground noise values, and the benefits of a Cassegrain telescope with respect to the mechanical stability, the control mode and the weight. It is optimized for broadband receiving system which usually have a wider flare angle of 65° compared to traditional feeds in geodetic VLBI like the RTW with 22°.

The main technical requirements and specifications for the TWIN telescopes are summarized in Table 1 below: 

**Table 1:** Major TWIN design criterions with explanations.

Criterion	Remarks
Diameter of main reflector *D* = 13.2 m	Trade-off between sensitivity and antenna speed as well as mechanical stability; RTW features an area of 314 m^2^, *i.e.*, 2.3 times larger than the TWIN main reflector; compensation by state of the art broadband receiving system and signal processing.
Ratio *f/D* = 0.29	The ratio of focal length *f versus* the diameter *D* of the main reflector is a central criterion of the ring focal design for a superior antenna illumination efficiency at a flare angle of about 65°.
Broadband capability *f* ≈ 1 to 40 GHz	Antenna system shall include L-band for possible future support of GNSS satellite tracking applications (currently concentrated at 1.2 to 1.5 GHz) and to fully cover Ka-band.
Surface finish better than 0.3 mm (RMS)	Lower RMS of surface finish required the higher the frequency in use is; actually obtained RMS values from quality control are significantly smaller than this requirement.
Motion velocities of 12 °/s in azimuth direction and 6 °/s in elevation	Essential requirement for VGOS antennas to scan more radio sources in the same time interval; RTW telescope has maximum speeds of only 4 °/s and 1.5 °/s, respectively.
Drive range ±270° in azimuth direction and 0–115° in elevation	Provides overlaps for efficient and quick steering to subsequent radio sources scheduled; also needed for VLBI satellite applications (un-interrupted tracking in regions close to zenith).
Balanced antenna design with high-performance bearings	Antenna will not move downwards in an instable position potentially leading to damage in case of power loss.
27 Bit Encoder with 0.0003° resolution	State-of-the resolution for precise radio source pointing.
Sub-reflector adjustable by a hexapod	Compensation of structural deformation of main and sub-reflectors due to gravity; total path length error is required to be below 0.3 mm.
Stable reference point with an axis offset of less than 5 arc-seconds	Important stability requirement to precisely reference the VLBI measurements geometrically.

The TWIN radio telescopes are particularly designed for the use as a geodetic measurement system. Since mechanical deformations directly deteriorate the accuracy of a VLBI measurement, the primary target was to develop an extremely stiff and stable radio telescope to get an optimal trade-off regarding the IVS requirements *versus* the budget available to the project. Since delays observed in VLBI can be directly related to length (and baseline vector) information, it is extremely important to minimize the “path length error”, which is the length deviation from an ideal line between the main reflector and the sub-reflector to the phase center of the feed horn. The path length error consists of a static part, caused by a phase center misalignment of the feed horn, and a dynamic part that is caused by the self-loaded deformation of the main reflector. The dynamic error was specified to be less than 0.3 mm. All elements, *i.e.*, the main reflector, the elevation cabin as well as the yoke and the concrete tower were optimized to keep the path length stable at all defined loading conditions (wind gust up to 40 m/s; dead load, temperature gradients up to 30 K). The mechanical construction of both reflectors was aligned to achieve a high degree of stiffness with the help of a special backing structure. Additionally a so called hexapod compensates the vertical shift of the main reflector focal line when moving the antenna in elevation direction.

### 2.2. Antenna and Reflectors

**Figure 2:**
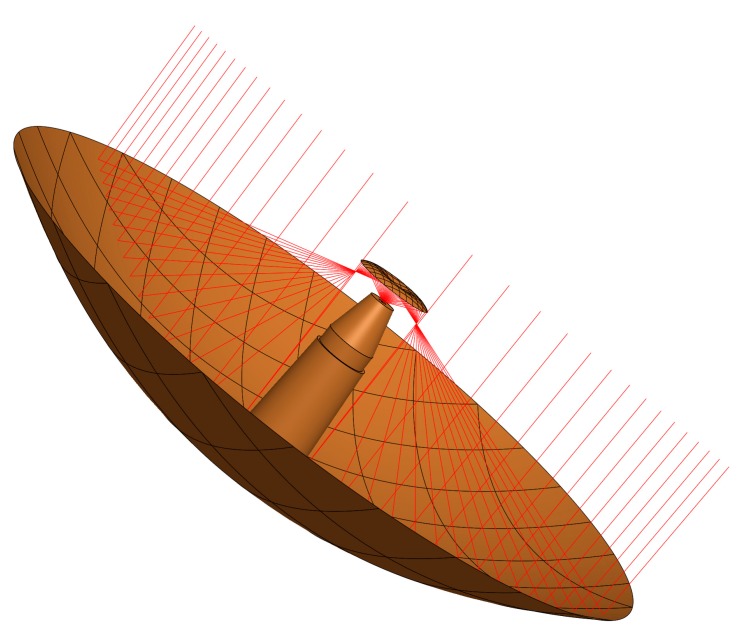
Path of rays for a ring focal antenna design. Image by courtesy of VERTEX Antennentechnik, Germany [12].

The ring focal configuration is up to now a rarely used receiving system in radio astronomy. The shape of the main reflector of this configuration is geometrically generated by the offset section of a parabola at the focal line which is rotated at the antenna’s axis of symmetry. The result is a main reflector that creates a ring-shaped first focal line before the sub-reflector. Figure 2 illustrates the path of rays for such a ring focal design. The actual diameter of the TWIN antenna main reflector is 13.2 m.

The advantage of this configuration is obvious: all microwaves that hit the area of the main reflector are directed to the most sensitive region of the feed system. This will enhance the aperture efficiency and minimize backward reflections at the sub-reflector. In contrary to the Cassegrain antenna configuration (as used for the RTW telescope), it is possible to mount the feed horn very closely to the sub-reflector to avoid any significant degradation of the illumination efficiency by blockage and reflected rays back from the sub-reflector. The advantage of this geometric configuration is a ray distribution yielding aperture efficiencies of more than 80%. Numbers like that are not possible using a standard dual reflector antenna without having a shaped reflector system.

A measure for the sensitivity of a radio telescope is the *System Equivalent Flux Density (SEFD)*, which represents the flux density of a fictitious radio source that has the same noise power level as the antenna system itself [13,14]:

(1) $$SEFD=\frac{2k\cdot T_{SYS}}{A_{EFF}}$$

Since the *SEFD* comprises the impact of the system noise temperature *T_SYS_* and the effective antenna area *A_EFF_*, it is a convenient way to compare different antenna systems (*k* = Boltzmann constant). A lower *SEFD* results in a better sensitivity of the system, which leads to a lower integration time at a given signal-to-noise-ratio (SNR):

(2) $$SNR=\frac{S(source)}{SEFD}\sqrt{t\cdot \text{ν}}$$

where *S(source)* is the flux density of the radio source in units of Jansky [Jy], *t* is the integration time in seconds [s] and ν is the bandwidth [Hz]. A wider bandwidth used for observing a radio source will yield a higher SNR. This is the reason for extending the bandwidth up to 1 GHz for each of the four bands to be recorded in the new VGOS radio telescopes compared to a maximum bandwidth of just 200 MHz in S-band for the existing RTW (X-band: 900 MHz).

**Figure 3:**
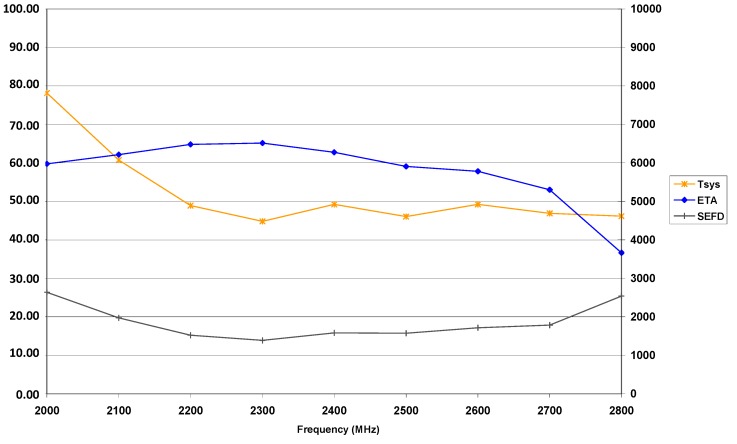
System noise temperature *T_SYS_* in [K], antenna efficiency *ETA* in [%] and *SEFD* (*y*-axis numbering on the right) in [Jy] of the TTW1 antenna in S-band.

**Figure 4:**
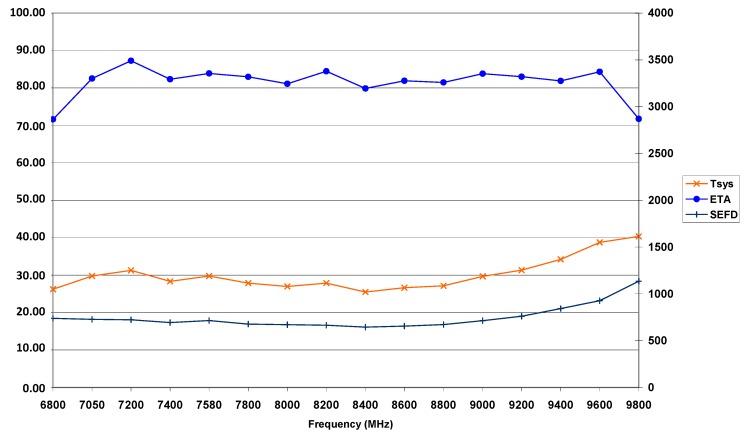
System noise temperature *T_SYS_* in [K], antenna efficiency *ETA* in [%] and *SEFD* (*y*-axis numbering on the right) in [Jy] of the TTW1 antenna in X-band.

Figure 3 and Figure 4 illustrate the sensitivity of the TTW1 antenna system in both S- and in X-band. VGOS specifications essentially require the antenna efficiency to be better or equal to 60% [6] which is fulfilled for most parts of the S-band spectrum and considerably exceeded for the complete X-band spectrum available from the tri-band feed horn. The SEFD is around 1500 to 1700 Jy in the S-band range intended for use, and around 750 Jy in X-band, which is a very encouraging result. Comparable values for the 2.3 times larger 20 m RTW are around 1200 Jy and 700 Jy, respectively.

### 2.3. Receiver System—Tri-Band Feed System

The tri-band feed [15,16] implemented in the TTW1 antenna is a major extension compared to current receiving systems used in geodetic VLBI: three different frequency bands are combined together in one feed. It consists of an interleaved coaxial waveguide system that enables a moderate bandwidth at S-Band (2.0 to 2.8 GHz) and a very wide bandwidth for both X- and Ka-Band (6.8 to 9.8 GHz as well as 27 to 34 GHz).

**Figure 5:**
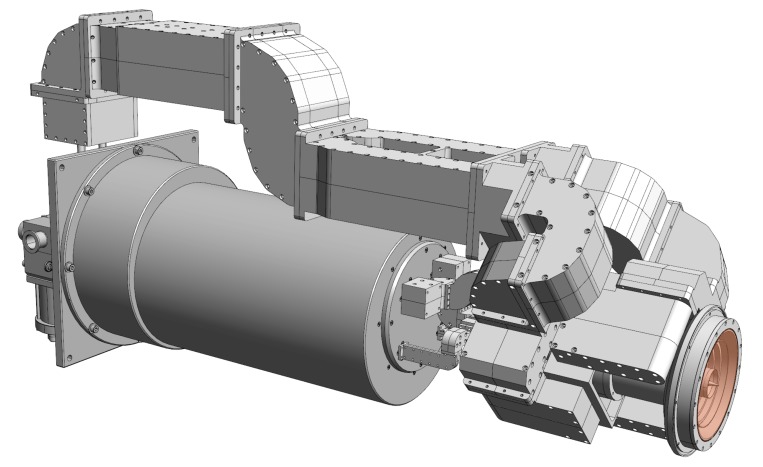
Tri-band feed horn; the part in marked in light brown constitute the main feed system, followed by the orthogonal mode transducer (OMT). The connected waveguides are combined by hybrid couplers to generate the LHCP and RHCP output signals. Also shown: The cylindrical Dewar with mounted cold head. Figure by courtesy of MIRAD AG, Switzerland.

The radio signal sampled in geodetic VLBI systems is quasar noise. We decompose this signal into a right (RHCP) and left hand circular polarized (LHCP) fraction. The signals received in the S- and X-band waveguide sections are extracted by radially mounted turnstile junctions and then combined to right and left hand circular polarized signals via a 90° hybrid. For the Ka-band section, a circular waveguide tube acts as a feed horn in the center of the tri-band feed. The Ka-band signal is directly divided into RHCP and LHCP waves by a septum polarizer. The connection to the Dewar via the front plane is only possible for X- and Ka-band due to the big mechanical extensions of the S-band waveguides. The S-band signal itself is connected by very short coaxial cables to the other end of the Dewar. A graphical illustration is given in Figure 5.

The Dewar is a high vacuum vessel, which contains the ultra-low-noise amplifiers (LNA), the precision directivity couplers and waveguide adapters. These are all cooled down to a particular temperature of 10 Kelvin. These cryogenic temperatures improve the inherent amplifier noise and phase stability of the received signals. A phase referencing signal (“phase calibration unit”, Pcal) is generated by a comb generator and injected in front of the LNA to define the phase reference at the inputs of the low-noise amplifiers [17]. The microwave outputs are connected by phase stable coaxial cables to the microwave down converter as depicted in the following section.

### 2.4. Initial and Final Down-Converters

The down-converter completes the receiving system, together with the feed horn, the cooling system (Dewar) and the backend. An initial version limited to S- and X-band in single polarization mode was developed for demonstration of first light capabilities and in order to perform legacy operations together with the 20 m RTW telescope. The final down-converter features tri-band capabilities in dual-polarization mode.

#### 2.4.1. S/X-Band Down-Converter

**Figure 6:**
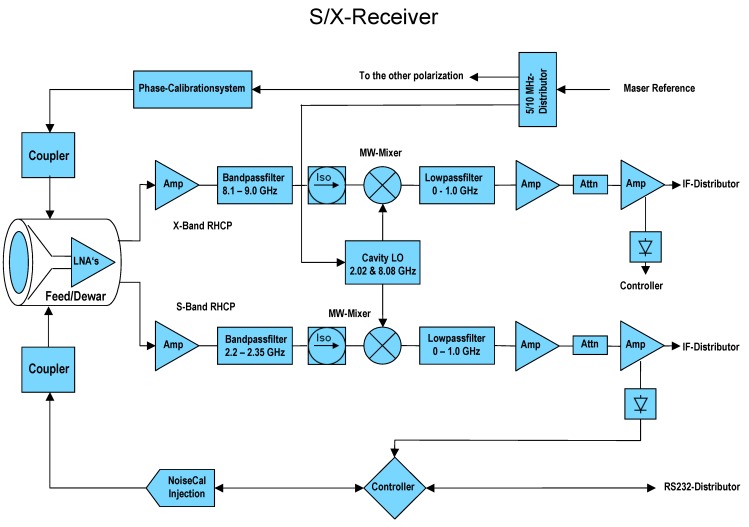
Block diagram of the initial TTW1 S/X-receiver developed for first light demonstration.

**Figure 7:**
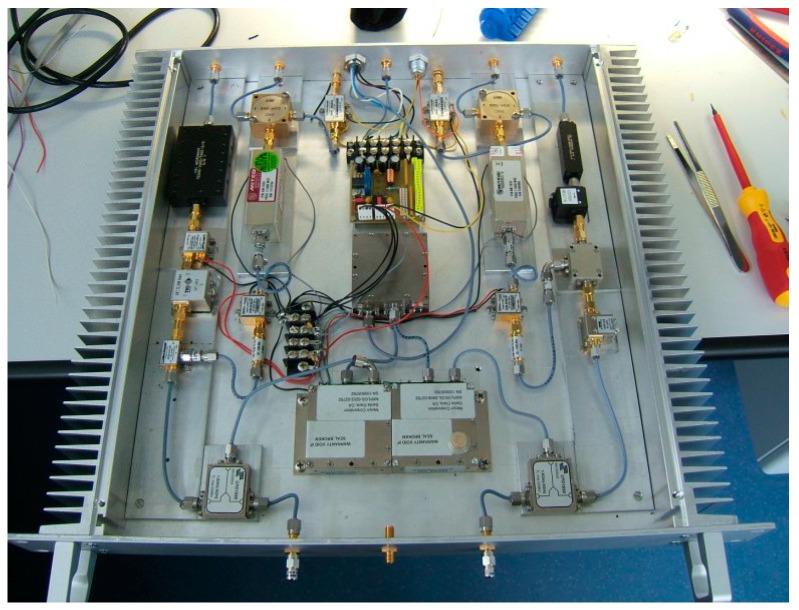
Photo of the Initial S/X-receiver.

The down conversion of the microwave signals of a standard geodetic receiver is done by the superheterodyne principal, where each frequency band is converted by a single mixer stage, followed by an intermediate frequency amplifier and a low-pass filter. An additional driver stage provides the high intermediate-frequency (IF) power level required to get sufficient power at the digital data acquisition system (DAQ), which records 16 dedicated 4 or 8 MHz wide frequency bands, see Figure 6 and Figure 7.

#### 2.4.2. Tri-Band Down-Converter

**Figure 8:**
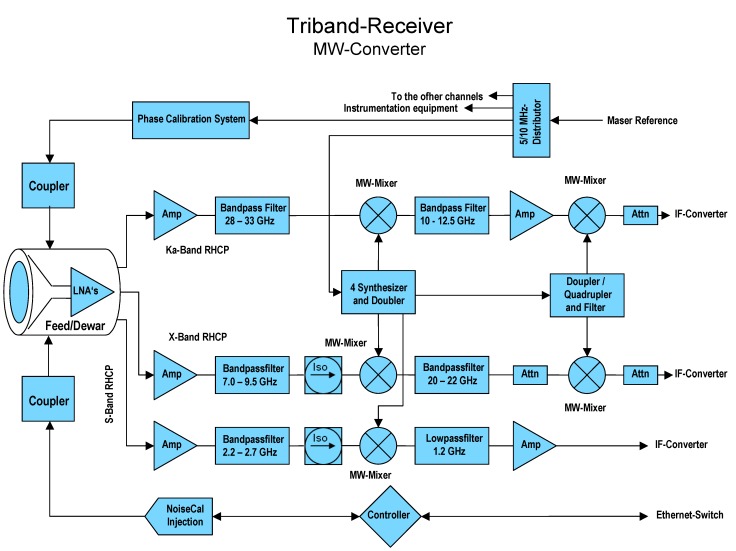
Tri-band down-converter block diagram for one selected polarization (RHCP).

**Figure 9:**
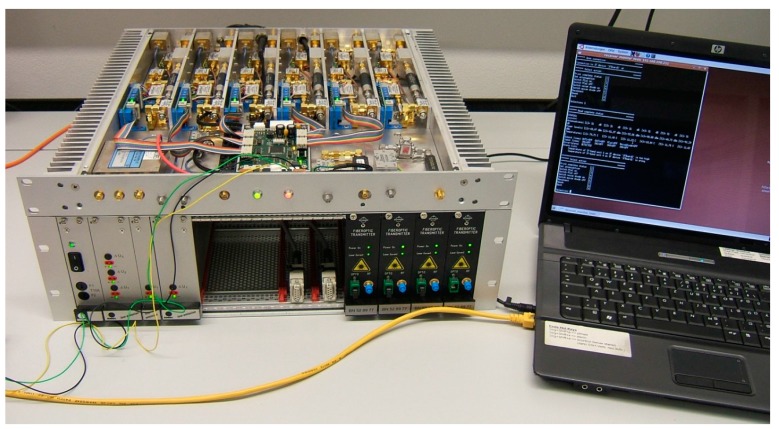
Picture of the IF-converter for the tri-band receiving system of TTW1.

The VGOS requirements [6] defined for VLBI receiving systems are very demanding: a continuous 2–14 GHz broad frequency spectrum is to be covered, and within this frequency range, an arbitrary selection of multiple bands with a bandwidth of 1 GHz wide band shall be selectable. This requirement triggered the development of a new concept for the microwave receiver, see Figure 8 and Figure 9. Additionally, both microwave polarizations (LHCP and RHCP) must be available, and implemented such that compatibility with the new linear polarized broadband system like the “Elevenfeed” for the second TWIN telescope TTW2 can be achieved. These requirements lead to a substantially more complex hardware for the new broadband receiver. In essence, it is necessary to use an up-down-converter for X-band and a down-down-converter for the Ka-band to transfer all bands into the requested IF-bands.

The key point is to achieve an effective and selectable down conversion of the microwave signals to different Nyquist zones, such as 0–512 MHz, 512–1024 MHz, 1024–1536 MHz and 1536–2046 MHz. A quadruple phase stable synthesizer is used as variable first local oscillator (LO) for the up-conversion. It is directly locked to a 100 MHz frequency reference derived from a hydrogen maser. This maser has a very high frequency stability of Δ*f/f* ≈ 2 × 10^−16^ over short terms of up to 1000 s which is essential for precise geodetic VLBI. The Quasar signal received by the antenna and amplified by the LNAs yields a power level of 10^−12^ W or less; the typical spectral flux density of the radio sources used in geodesy is around 0.5 to 1.0 Jy (1 Jy = 10^−26^ W/m² Hz). Hence, a receiver with an excellent spurious and crosstalk rejection (>60 dB) had to be designed.

#### 2.4.3. Intermediate-Frequency (IF) Converter

**Figure 10:**
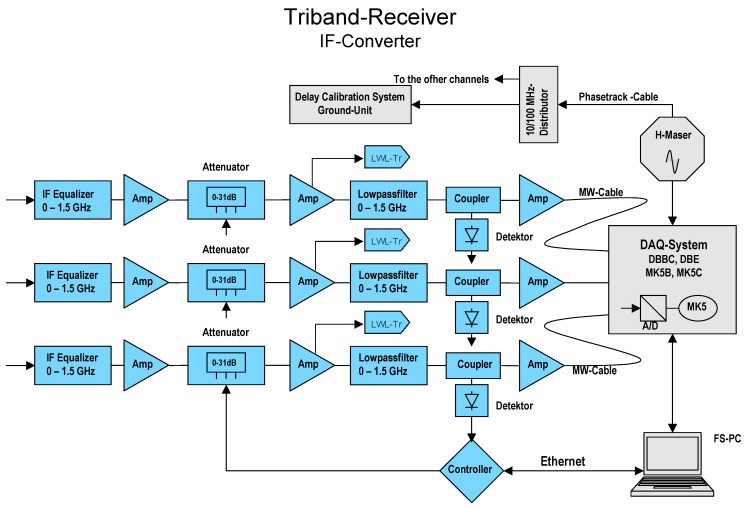
IF-converter scheme and DAQ (data acquisition) system.

The IF-amplifier stage developed for TTW1 adjusts the down-converted microwave signal to yield a high signal integrity at the data acquisition (DAQ) system, see Figure 10. The main goal was the design of a universally useable module for all the necessary Nyquist frequency bands. Therefore, the IF-bandwidth was kept as high as possible (up to 1.5 GHz) in order to be able to supply all necessary IF-signals which the data acquisition system can support. The selection of the desired Nyquist zone is done in the DAQ-System itself. The IF-stage includes an improved broadband equalizer that compensates the frequency-dependent cable attenuation in the microwave receiver. Two amplifier stages were combined with a variable attenuator to get a level-adjustment at a magnitude of about 30 dB. A low-pass filter and a medium power amplifier, together with an RMS-to-DC converter, generates the suitable signal level at the input of the low temperature coefficient coaxial cables. An additional connection to an optical fiber transmitter is also foreseen. The power measurement is used to calculate the system temperature for each frequency band separately. All coaxial cables are terminated by an IF distribution system before reaching the DAQ-System (not shown in Figure 10).

### 2.5. Backend

The main requirements for the recording system are defined by the previously stated number of signals to be received (up to three for S/X/Ka-band with a bandwidth of up to 1 GHz each) and the number of polarizations (LHCP and RHCP). This yields six radio frequency bands to be converted down to intermediate frequency (IF) for registration—via a digital base-band conversion process—by the recording system.

The recording of the received noise signals in the IF bands is realized by a digital base-band converter DBBC-2 [18]. This device samples the analog signals of up to four IF-bands synchronously with an amplitude resolution of 8 bits and a sampling frequency of 1 GSps (giga-samples per second). The DBBC is synchronized with the reference frequency (10 MHz) of the hydrogen maser. The statements in Section 2.4.2 apply to the DBBC: The IF frequency range is divided into frequency windows as mentioned in Section 2.4.2 (2nd paragraph) with help of frequency band filters. This warrants an alias-free signal processing of the IF bands sampled. The DBBC configuration is flexible: up to 16 freely selectable frequency channels can be recorded with a bandwidth of 0.5–32 MHz each, either in direct down-converter mode (DDC) or using the polyphase filterbank mode (PFB). The latter can coherently record a frequency window as broad as 480 MHz. The DBBC-2 can manage two IF-inputs in PFB mode and provide outputs on the two available VLBI standard interface (VSI) ports.

A number of experiments were performed in the period between July 2014 and March 2015 to test the TTW1 performance. Regarding the configuration, two types of experiments were carried out with different setups as outlined in the following tables. In essence, the setup of the 24 h experiments (Whisp1/2 and Euro130, Table 2; indicated by a “W” or “E” in the corresponding tables in analysis Section 4) differs from the Intensive experiments (Table 3; indicated by an “I” in the corresponding tables in the analysis Section 4).

**Table 2:** Setup for the sessions Whisp1, Whisp2 and Euro130 in direct down-converter mode (DDC mode; LO = local oscillator [frequency], IF = intermediate frequency).

Frequency Band	Channel	Radio Frequency	LO	IF	Channel Bandwidth	Upper/Lower Sideband	Sample Rate	Channel Spacing	Total Span
		(MHz)	(MHz)	(MHz)	(MHz)		(MSps)	(MHz)	(MHz)
X-Band	1	8210.99	8080	130.99	4	USB/LSB	8 + 8		
X-Band	2	8220.99	8080	140.99	4	USB	8	10	
X-Band	3	8250.99	8080	170.99	4	USB	8	30	
X-Band	4	8310.99	8080	230.99	4	USB	8	60	
X-Band	5	8420.99	8080	340.99	4	USB	8	110	
X-Band	6	8500.99	8080	420.99	4	USB	8	80	
X-Band	7	8550.99	8080	470.99	4	USB	8	50	
X-Band	8	8570.99	8080	490.99	4	USB/LSB	8 + 8	20	360
S-Band	9	2212.99	2020	192.99	4	USB	8		
S-Band	10	2227.99	2020	207.99	4	USB	8	15	
S-Band	11	2237.99	2020	217.99	4	USB	8	10	
S-Band	12	2267.99	2020	247.99	4	USB	8	30	
S-Band	13	2287.99	2020	267.99	4	USB	8	20	
S-Band	14	2292.99	2020	272.99	4	USB	8	5	80
Total Data Rate							128		

**Table 3:** Setup for the “Intensive” sessions in direct down-converter mode (DDC mode; LO = local oscillator [frequency], IF = intermediate frequency).

Frequency Band	Channel	Radio Frequency	LO	IF	Channel Bandwidth	Upper/Lower Sideband	Sample Rate	Channel Spacing	Total Span
		(MHz)	(MHz)	(MHz)			(MSps)	(MHz)	(MHz)
X-Band	1	8210.99	8080	132.99	8	USB/LSB	16 + 16		
X-Band	2	8220.99	8080	172.99	8	USB	16	40	
X-Band	3	8250.99	8080	272.99	8	USB	16	100	
X-Band	4	8310.99	8080	432.99	8	USB	16	160	
X-Band	5	8420.99	8080	652.99	8	USB	16	220	
X-Band	6	8500.99	8080	772.99	8	USB	16	120	
X-Band	7	8550.99	8080	832.99	8	USB	16	60	
X-Band	8	8570.99	8080	852.99	8	USB/LSB	16 + 16	20	720
S-Band	9	2212.99	2020	205.99	8	USB	16		
S-Band	10	2227.99	2020	225.99	8	USB	16	20	
S-Band	11	2237.99	2020	245.99	8	USB	16	20	
S-Band	12	2267.99	2020	275.99	8	USB	16	30	
S-Band	13	2287.99	2020	325.99	8	USB	16	50	
S-Band	14	2292.99	2020	345.99	8	USB	16	20	140
Total Data Rate							256		

#### 2.5.1. Digital Signal Representation

The digital representation of the analogue input is realized via parallel processing signal structures, numerical oscillators (NCO) and digital filters implemented in FPGA-chips. The signals are processed by down-conversion into separate base-band channels, more precisely into 16 base-band channels in DDC mode and 15 base-band channels in PFB mode. The sample rate in each base-band channel is fixed to 32 MSps and the amplitude resolution is two bits. The adjustment of the necessary base-band channel bandwidth and the distinction between 1 and 2 bit sampling is done in the setup of the Mark 5B+ data recorder by setting a decimation factor and an appropriate track selection mask. The sampled signals are of real representation (not complex). The total data rate is given by the data rate of each base-band and the amplitude resolution. Thus, the recorded total data rate of a VLBI observation can be chosen by adjusting these parameters (e.g., 256 Mbps for an Intensive experiment). Using the maximum of two VSI outputs, a maximum data rate of 2 GSps can be reached. One standard VLBI recording system of type Mark 5B+ [19,20] is connected to each output of the DBBC. The sample clock rate of each base-band channel is decimated to 32 MHz at the VSI output.

The classical VLBI data recording system of the 20 m RTW for S- and X-band (single polarization, RHCP) splits the X-band into 10 channels and the S-band signal into six channels. The IF bandwidth is 200 MHz in S-band and 900 MHz in X-band. A maximum overall data rate of 1 GSps with all these 16 base-band channels in use and a data rate of 32 MHz per base-band as well as an amplitude resolution of 2 bits is obtained.

#### 2.5.2. Full Recording Configuration for TTW1

**Figure 11:**
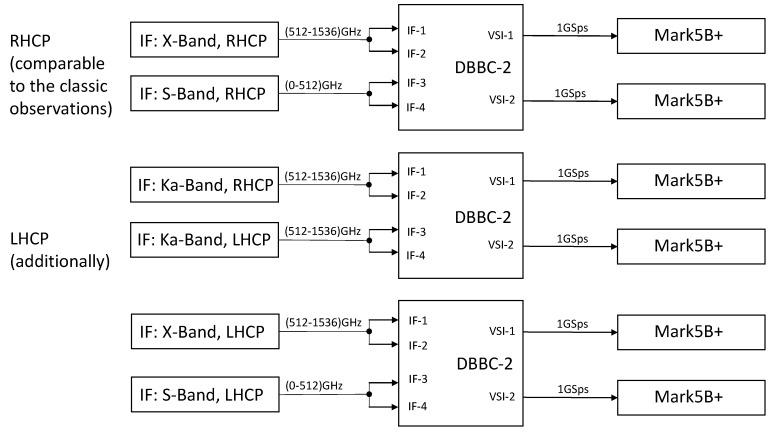
Illustration of the TTW1 base-band conversion and recording system.

The final configuration of the recording system for TTW1 in full DDC mode comprises three DBBCs with 6 Mark 5B+ data recorders, see Figure 11. The data are processed and sampled at a maximum speed of 6 GSps. In contrast, the full sampling of 1 GHz broad signals in PFB mode will require six DBBCs and 12 Mark 5B+ units, since one DBBC-2 will be required for processing of each single IF band. Consequently, the data rate would double to 12 GSps which is the limit in this TTW1 configuration design, *i.e.*, without using alternative recording techniques.

### 2.6. Operating and System Monitoring Software

The monitoring and control software of the new TWIN radio telescopes follows the approach to connect distributed software agents. Software agents are intelligent, autonomous units, which split complex technical environments into manageable tasks in the software system. The agents are able to make own decisions for their specific scenarios and environments to meet their delegated objectives [21,22]. Each device to be controlled or monitored follows the design of such agents. It collects data from the according hardware. This information is evaluated to make scheduling and controlling decisions using predefined metrics with defined guidelines and parameters. The process results in the commanding of new activities on the hardware. Therefore critical, security relevant determinations are processed directly in these agents, which optimize the processing workflow. It is the basis for the used monitoring and control infrastructure.

The communication between and with the agents uses a specially developed communication middleware. The background are Remote Procedure Calls (RPC) of Sun Microsystems that build up an abstraction layer, which extends functionalities of the communication hardware and process in a standardized, safe and homogenized form ([23], p. 2). The basis is a software generator “idl2rpc.pl”, which was especially developed in Wettzell, using the programming language Perl. It reads an interface definition and creates all functionalities and services of a server and client system, where the client request services from a service offering server.

The software components created are integrated into the NASA Field System [24], which is globally used to operate VLBI radio telescopes. It is the layer for the controlling and monitoring as a central control system. The system organizes the centralized controlling of observations with a predefined schedule file. Additionally, it offers several programs to deal with data or error situations or to plot data. The NASA Field System even defines its own command line interface to realize scripts for VLBI-observation schedules.

As an extension to the Field System the remote control software “e-RemoteCtrl” [25] is used to enable a global remote control and automation. The software was partly developed within the EU-project “Novel EXplorations Pushing Robust e-VLBI Services (NEXPReS)”. It allows a secure access of several, globally distributed observers to a monitoring and control server, using a tunnel-ing technique on the basis of the Secure Shell. The server can retrieve data or inject commands directly to the NASA Field System, while the access is restrictively defined with user rights and access roles. The server is the final decision-making instance. The software “e-RemoteCtrl”, which was designed and developed at the Geodetic Observatory Wettzell, is already globally in use. Among other things, it is also the backbone for the operation of the remotely controlled telescopes of the geodetic and astronomical VLBI-network AuScope in Australia [26].

## 3. Baseline Correlation

This section specifically deals with the VLBI data correlation using the DiFX software correlator [27]. These tests consisted of running VLBI experiments using a geodetic dual-band setup as detailed in Section 2.5, and thereafter performing the cross correlation [28]. During the data analysis after the correlation process, we could establish the quality of the TTW1 data with respect to other antennas that are regularly used and are known to be reliable and well calibrated (*i.e.*, the RTW and the Tsukuba antenna in Japan). Note that a performance assessment regarding the baseline results in terms of coordinate differences is given in the following Section 4.

### 3.1. X-Band Correlation Results

**Figure 12:**
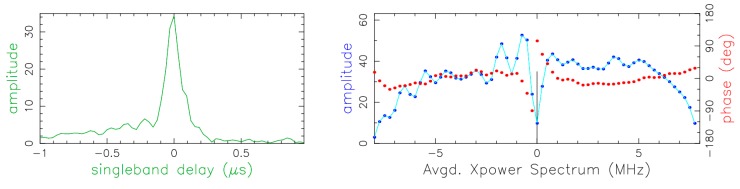
Correlator output for the baseline Tsukuba/TTW1 at 8.4 GHz. (**left**) Single band delay (SBD); (**right**) Fourier transform of the SBD, *i.e.*, averaged power spectrum. The red line corresponds to the phase and the blue line to the amplitude of the fringe visibilities.

Figure 12 shows one scan on the baseline between TTW1 and Tsukuba in X-band. The left side shows the single band delay (SBD) in μs, *i.e.*, the amplitude output of the correlator integrated over the scan duration. On the right the averaged power spectrum is plotted that is the Fourier transform of the SBD into the frequency domain (red = phase, blue = amplitude). The peak of the SBD is well defined and centered at zero as one would expect after the correlation and the residual delay correction. To have good fringes one expects to see the phase (red line) well aligned, as is visible in the plot. The signal-to-noise ratio (SNR) obtained from this scan was 101.

We then calculated the theoretical SNR for the source observed within the scan considered (radio source ICRF J175132.8 + 093900, flux density at X-band is about 3 Jy). Given the effective sampled bandwidth of 64 MHz (8 BBC channels each 8 MHz wide) and the integration time of 15 s, the *a priori* SNR is about 100. This operation was repeated for all the tests for both frequency bands. The differences between the *a priori* and the measured *a posteriori*) SNR are statistically not significant indicating that the TTW1 performed well.

### 3.2. S-Band Correlation Results

**Figure 13:**
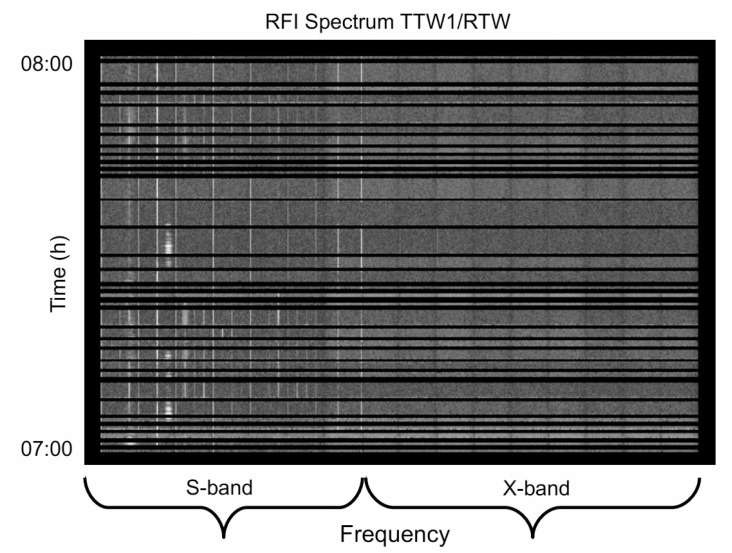
RFI spectrum common to TTW1 and RTW (ordinate: time in hours; abscissa: frequency). The left part of the picture depicts the S-band where one can see vertical white lines that represent radio frequency interference.

The band at 2.3 GHz (S-band) is contaminated by radio frequency interference (RFI), typically caused by telecommunication satellites. Such contamination is present on almost all the baselines. However, on the long baselines, which are the ordinary scenario in VLBI, the correlator fringe rotator wraps the RFI phases many times within one integration period (1 s). In most cases, this reduces the amplitudes of the RFI close to zero. In contrast, on the very short baselines between TTW1 and RTW, the correlator fringe rotator does not wind up the phases of the RFI, hence the RFI is not attenuated and corrupts the data. Figure 13 shows the RFI spectrum common to the TTW1 and RTW as obtained using the astronomical image processing system AIPS [29,30].

The abscissa is the frequency and the ordinate is the time in hours. The strong vertical lines on the left side of the plot are caused by RFI. Some lines are thin in frequency and are present for the whole experiment (possibly signals broadcast from telecommunication satellites). Some others appear only for a certain period of time (possibly nearby interference).

**Figure 14:**
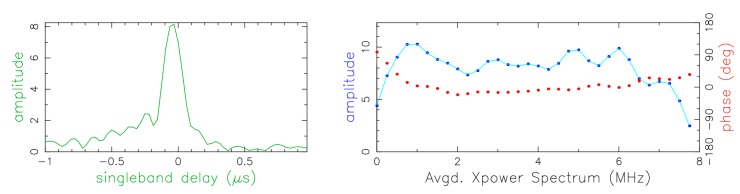
Correlator output for the baseline Tsukuba/TTW1 at 2 GHz. The radio source is ICRF J172727.6 + 453039.

From the RFI analysis we can conclude that the observables obtained over the short baseline TTW1/RTW are in parts unusable. In contrast, those obtained on long baselines between TTW1 and—in this case—Tsukuba, are well-usable for further geodetic analysis, also see Figure 14: if RFI had been present, it would have been visible as spikes on the amplitude part of the cross-correlation spectrum, which instead is clean, and the signal-to-noise level is appropriate for S-band measurements.

## 4. Initial Processing Results

The initial results from 26 experiments carried out in between June 2014 and March 2015 are presented in this section with a focus on coordinate precision at the local level. For this purpose, the 123 meter baseline between the “old” RTW telescope and the “new” TTW1 telescope is analyzed. The advantage of this method, which in fact is very short baseline interferometry rather than very long baseline interferometry as commonly practiced, is that a number of problematic error sources virtually cancel out over short baselines, in particular atmospheric propagation delays. In contrast, local RFI is a significant problem. We assume that RFI is mainly present in S-band and hardly in X-band as illustrated in Section 3. Consequently, we analyze S- and X-band separately. Finally, the reference points of all instruments at the observatory are regularly measured via a precise local network survey, so reference data are available including the new TTW1. The reference point of all VLBI telescopes at the Observatory is defined as the intersection point of the azimuth and the elevation axes. The disadvantage is that the RTW telescope sets the limits: Only conventional S-band and X-band data can be compared in the analysis (also see Section 2.5.1).

Note that 23 experiments are of the “Intensive” type. The associated backend setup is detailed in Table 3. The setup for the three 24-h experiments is given in Table 2. Twenty four experiments were carried out using the S/X down-converter (Section 2.4.1) and two initial ones with the tri-band down-converter (Section 2.4.2). The digital backend in use is a DBBC-2 with results being presented in Table 4 (S-band) and Table 5 (X-band). Additionally, six experiments were sampled with an ADS3000+ in parallel, and are presented separately in Table 6 (S-band) and Table 7 (X-band).

### 4.1. Data Analysis

Data analysis is primarily carried out using an in-house software developed for data quality analysis as well as local network adjustment of radio telescope data collected over short distances. In addition, the Calc/Solve software package [31,32] is used for processing selected experiments for purposes of comparison. This is a well-known package and wide-spread within the geodetic VLBI community.

#### 4.1.1. Short-Baseline in-House Software of Geodetic Observatory Wettzell

The basic observation equation for the group delay τ*_G_* between RTW and TTW1 to radio source *q* reads [3,4]:

(3) $${\text{τ}}_{G}=-\frac{1}{c}\cdot \left(bx\cdot \text{cos }t_{q}\cdot \text{cos }{\text{δ}}_{q}+by\cdot \text{sin }t_{q}\cdot \text{cos }{\text{δ}}_{q}+bz\cdot \text{sin }{\text{δ}}_{q}\right)$$

where *c* is the speed of light, *bx*, *by*, *bz* are the geocentric coordinate differences between RTW and TTW1 (baseline components, the key parameters of interest in our analysis), *t_q_* is the Greenwich hour angle to radio source *q* (a function of its right ascension α and Greenwich apparent sidereal time GAST) and δ*_q_* is the declination of the radio source.

The RTW and TTW1 telescopes are currently connected to individual hydrogen masers. A common oscillator base is planned for the near future, but as long as this is not the case, the differences in clock behavior need to be compensated. This is done via a clock error polynomial, so the observation equation is expanded as follows:

(4) $${\text{τ}}_{G}=-\frac{1}{c}\cdot \left(bx\cdot \text{cos }t_{q}\cdot \text{cos }{\text{δ}}_{q}+by\cdot \text{sin }t_{q}\cdot \text{cos }{\text{δ}}_{q}+bz\cdot \text{sin }{\text{δ}}_{q}\right)+\sum_{i=0}^{n}a_{i}\cdot \Delta t^{i}$$

where *a_i_* are the clock error coefficients and Δ*t* is the time difference between the current observation epoch and the initial observation epoch (the first data records collected). The polynomial is a linear function (*n* = 1) for experiments that last about one hour and is expanded accordingly for longer experiments. Both short experiments (1 h) and long experiments (24 h) have been conducted within the scope of this study.

The baseline components between both telescopes are precisely known from terrestrial surveying. In this paper we will focus on the 3D distance d_3D_ between the two telescopes which is:

(5) $$d_{3D}=\sqrt{bx^{2}+by^{2}+bz^{2}}\approx 123.3070\text{ m}$$

The accuracy (standard deviation) of this distance is σ < 0.7 mm. Actual positioning results from data analysis are compared to this reference value.

Several reductions and corrections are usually applied to observations, radio source positions or antenna positions [2,4]. Not all of such possible refinements are taken into consideration in this initial study. The radio source positions are reduced to the current observation epoch taking into account reductions due to precession, nutation and annual aberration. A navigation grade algorithm is applied for the in-house software [33] and should feature arc-second accuracy sufficient for the initial data analysis in this study. The reduction from the Conventional International Origin (CIO) to the instantaneous pole as well as equator and Greenwich meridian are carried out with the help of the pole position and Earth rotation data from the International Earth Rotation and Reference Systems Service (IERS).

Possible corrections due to antenna structure are not applied. Such effects have not been investigated in case of the new TWIN telescopes, and are not considered to be of major concern here. Tropospheric and ionospheric propagation delays essentially cancel out over the short baseline of just 123 m, because both troposphere as well as ionosphere are spatially correlated. However, note that a small residual tropospheric delay error will be present in the results due to the height difference of 3.4 m between the two reference points. A future version of the analysis software is planned to take this effect into consideration. Earth tide reductions are not applied for the same reason. Radio source structure corrections are not applied.

The retarded baseline reduction τ_RTB_ accounts for the fact that both antennas are not motionless during the signal travel time from one telescope to the other. In fact, the Earth is rotating, so a reduction essentially accounting for diurnal aberration [1,4] is applied:

(6) $${\text{τ}}_{RTB}=-\frac{\text{ω}\cdot {\text{τ}}_{G}}{c}\cdot \left(Y_{2}\cdot \text{cos }t_{q}\cdot \text{cos }\delta_{q}-X_{2}\cdot \text{sin }t_{q}\cdot \text{cos }\delta_{q}\right)$$

where *X*_2_ and *Y*_2_ are the two coordinate components of the second telescope in the processing chain (RTW), ω is the angular velocity of the Earth and τ_G_ is the group delay observation (not corrupted by clock errors). Although the baseline between RTW and TTW1 is small, this reduction can amount to a couple of tens of millimeters. So, it is taken into consideration to avoid accuracy losses.

#### 4.1.2. Calc/Solve Parametrization Setup

Selected experiments were analyzed independently by the VLBI group at the Institute of Geodesy and Geoinformation of the University of Bonn. The solutions were generated with the VLBI analysis software package Calc/Solve applying least squares adjustments. Based on observations involving several VLBI telescopes and radio sources, different parameter types, such as coordinates of stations, Earth orientation parameters or radio source positions as well as clock and tropospheric model parameter corrections can be estimated.

In this initial study, a typical parametrization setup for independent sessionwise VLBI data analysis was chosen. The telescope coordinates were estimated with the positions of the radio sources fixed to the International Celestrial Reference Frame ICRF2 [34]. The solution was performed using hard constraints including no-net-translation (NNT) and no-net-rotation (NNR) conditions [35] with three equations each in order to eliminate the datum defect. The clock parameters were modeled by a quadratic polynomial with additional continuous piecewise linear functions (CPWLF) with a temporal resolution of 60 min. The zenith wet delays (ZWD) as the wet part of the tropospheric delay were estimated as one offset parameter and no troposphere gradients were considered in this initial study. The Vienna Mapping Function 1 (VMF1) was used for tropospheric mapping [36].

### 4.2. Experiments

The TTW1 telescope performed 24 experiments in parallel with the RTW between June 2014 and February 2015 using the initial S/X-band receiver as sketched in Section 2.4.1. In addition, two other experiments were observed using the brand-new tri-band receiver (Section 2.4.2). These experiments were conducted on 23 February 2015 and 2 March 2015, totaling in 26 experiments during the TTW1 commissioning phase so far. The complete list of the S-band results is given in Table 4. Most of these were standard experiments conducted routinely by RTW for the International VLBI Service for Geodesy and Astrometry and accompanied by TTW1. The majority of the experiments is of type “I”—“Intensives” [37,38]. These experiments are one hour long, and the target is the determination of ΔUT1 = UT1 − UTC. Although this type of experiment is not optimized for baseline vector determination, it is still possible to derive the vector between TTW1 and RTW. For our analysis we also used one IVS-Euro session [39], type “E”, and two other dedicated experiments (Whisp, “W”). These sessions were 24 h long and by far contain the largest number of observations between TTW1 and RTW.

Note that the TTW1 phase calibration unit [17] was disabled during the majority of the experiments (only RTW phase calibration was on), because the fringe rate on such a short baseline is low, hence it corrupts the cross-correlation leaving visible only the tones of the phase calibration unit. This may introduce an additional error.

The default backend for TTW1 is the DBBC-2, Section 2.5. A few experiments were also recorded using both a DBBC-2 and a Japanese ADS3000+ [40]. This was implemented to investigate certain technical problems in S-band which, in the end, turned out to be radio-frequency interference.

### 4.3. Discussion of Results

Table 4 and Table 5 show the S-band results of the VLBI group delay analysis, and Table 6 and Table 7 show the X-band results. The type of experiment is explained in Section 4.2 (I = Intensive, 1 h; E = Europe, 24 h; W = Whisp, 24 h). The number of scans in the result tables is identical to the number of group delays observed (successfully correlated) over the corresponding baseline. The actual number of delays used in the least-squares adjustment process, solving for the baseline components as well as the clock polynomial, is given in column “accepted scans”. Observations classified as corrupted or unhealthy are summarized in column “rejected scans”; these scans did not become part of the adjustment procedure. A conventional outlier detector based on empirical precision estimates was used here [41].

The standard deviation of unit weight before the start of the adjustment (“σ_0_ *a priori*”) is computed from the standard deviations of the group delays as determined from the correlation process. The standard deviation of unit weight after completion of the adjustment procedure (“s_0_ *a posteriori*”) is computed from the residuals taking the individual weight of the observations into account. It is an empirically derived number. *A priori* and *a posteriori* standard deviations of unit weight should have a similar magnitude. If the *a posteriori* values are substantially higher than the *a priori* ones then either the *a priori* information is too optimistic (*i.e.*, the data quality is poorer than expected), or there are problems in the data modeling (e.g., an inappropriate compensation of clock error drift—actually not observed here). The variances of the parameters are scaled by the *a posteriori* variance of unit weight.

**Table 4:** Results obtained from S-band group delays using the DBBC backend. The last two experiments were measured using the tri-band down-converter (see Section 2.4.2).

Date	Type	Scans	Accepted Scans		Rejected Scans		σ_0_ *a Priori*		s_0_ *a Posteriori*		3D Difference
							(ps)	(mm)	(ps)	(mm)	(mm)
2 June 2014	I	11	11	100%	0	0%	124	37	11,079	3321	−2478.9
16 June 2014	I	13	11	85%	2	15%	88	26	70	21	−84.7
23 June 2014	I	20	20	100%	0	0%	89	27	245	74	54.3
7 July 2014	I	18	16	89%	2	11%	104	31	158	47	22.5
21 July 2014	I	15	15	100%	0	0%	95	29	207	62	0.7
20 August 2014	E	213	204	96%	9	4%	124	37	185	55	−0.6
27 August 2014	W	556	531	96%	25	4%	159	48	298	89	13.7
22 September 2014	I	12	9	75%	3	25%	148	44	111	33	151.1
29 September 2014	I	9	8	89%	1	11%	95	28	821	246	−381.7
6 October 2014	I	11	7	64%	4	36%	83	25	20	6	−377.8
20 October 2014	I	10	6	60%	4	40%	114	34	3	1	100.4
23 October 2014	W	927	739	80%	188	20%	374	112	552	165	−21.4
3 November 2014	I	21	17	81%	4	19%	142	42	427	128	−85.0
10 November 2014	I	12	6	50%	6	50%	140	42	10	3	−57.0
17 November 2014	I	19	16	84%	3	16%	137	41	202	61	−40.4
24 November 2014	I	13	11	85%	2	15%	76	23	197	59	−151.3
1 December 2014	I										*rejected*
22 December 2014	I	18	18	100%	0	0%	99	30	182	55	53.0
29 December 2014	I	18	17	94%	1	6%	117	35	371	111	−12.4
5 January 2015	I	21	16	76%	5	24%	141	42	784	235	−260.7
12 January 2015	I	13	13	100%	0	0%	135	40	33,961	10,181	3959.3
19 January 2015	I	17	17	100%	0	0%	117	35	486	146	155.2
26 January 2015	I	20	16	80%	4	20%	86	26	163	49	68.3
2 February 2015	I	22	19	86%	3	14%	83	25	238	71	−84.0
23 February 2015	I	25	18	72%	7	28%	75	23	138	41	9.8
2 March 2015	I	17	17	100%	0	0%	110	33	25,533	7655	2989.8

**Table 5:** Results obtained from X-band group delays using the DBBC backend. The last two experiments were measured using the tri-band down-converter (see Section 2.4.2).

Date	Type	Scans	Accepted Scans		Rejected Scans		σ_0_ *a Priori*		s_0_ *a Posteriori*		3D Difference
							(ps)	(mm)	(ps)	(mm)	(mm)
2 June 2014	I	25	19	76%	6	24%	11	3	13	4	−0.8
16 June 2014	I	24	24	100%	0	0%	7	2	13	4	−2.3
23 June 2014	I	22	22	100%	0	0%	7	2	11	3	−1.7
7 July 2014	I	22	22	100%	0	0%	9	3	10	3	0.4
21 July 2014	I	19	19	100%	0	0%	10	3	13	4	−2.0
20 August 2014	E	224	222	99%	2	1%	10	3	14	4	1.1
27 August 2014	W	661	653	99%	8	1%	15	4	24	7	−1.8
22 September 2014	I	20	20	100%	0	0%	14	4	19	6	4.4
29 September 2014	I	28	28	100%	0	0%	14	4	17	5	7.9
6 October 2014	I	24	20	83%	4	17%	7	2	10	3	−7.9
20 October 2014	I	21	21	100%	0	0%	11	3	14	4	−1.6
23 October 2014	W	999	947	95%	52	5%	18	5	35	11	−0.8
3 November 2014	I	27	27	100%	0	0%	7	2	12	3	−0.6
10 November 2014	I	30	30	100%	0	0%	15	4	15	5	−4.0
17 November 2014	I	22	21	95%	1	5%	10	3	12	3	−4.0
24 November 2014	I	35	35	100%	0	0%	11	3	12	4	−1.5
1 December 2014	I	24	24	100%	0	0%	9	3	10	3	1.8
22 December 2014	I	29	28	97%	1	3%	9	3	10	3	−1.1
29 December 2014	I	32	32	100%	0	0%	9	3	14	4	0.2
5 January 2015	I	33	30	91%	3	9%	14	4	16	5	−0.2
12 January 2015	I	25	22	88%	3	12%	30	9	33	10	−2.4
19 January 2015	I	25	25	100%	0	0%	9	3	12	4	−1.2
26 January 2015	I	32	29	91%	3	9%	17	5	19	6	7.4
2 February 2015	I	31	28	90%	3	10%	11	3	12	4	1.6
23 February 2015	I	29	27	93%	2	7%	9	3	19	6	8.2
2 March 2015	I	17	16	94%	1	6%	20	6	25	7	20.8

**Table 6:** Results obtained from S-band group delays using the ADS3000+ backend.

Date	Type	Scans	Accepted Scans		Rejected Scans		σ_0_ *a Priori*		s_0_ *a Posteriori*		3D Difference
							(ps)	(mm)	(ps)	(mm)	(mm)
7 July 2014	I	19	19	100%	0	0%	122	37	132	40	155.6
21 July 2014	I	19	16	84%	3	16%	105	32	176	53	−42.5
27 August 2014	W	564	532	94%	32	6%	155	47	294	88	15.7
22 September 2014	I	14	12	86%	2	14%	133	40	274	82	134.8
24 November 2014	I	26	24	92%	2	8%	114	34	264	79	−88.9
1 December 2014	I	20	19	95%	1	5%	124	37	145	44	116.1

**Table 7:** Results obtained from X-band group delays using the ADS3000+ backend.

Date	Type	Scans	Accepted Scans		Rejected Scans		σ_0_ *a Priori*		s_0_ *a Posteriori*		3D Difference
							(ps)	(mm)	(ps)	(mm)	(mm)
7 July 2014	I	22	22	100%	0	0%	9	3	9	3	−1.2
21 July 2014	I	23	19	83%	4	17%	9	3	11	3	0.3
27 August 2014	W	662	641	97%	21	3%	14	4	26	8	−2.0
22 September 2014	I	20	19	95%	1	5%	14	4	12	4	−9.1
24 November 2014	I	35	34	97%	1	3%	11	3	10	3	−0.2
1 December 2014	I	24	24	100%	0	0%	8	2	9	3	0.7

The differences of the estimated spatial distance between the two telescopes and the reference value from precision local surveying in the last column of Table 4, Table 5, Table 6 and Table 7 indicate the level of agreement.

#### 4.3.1. S-Band Results

As already outlined in the correlation section of this paper, RFI is present in S-band and destroys the signal on short baselines. The old RTW telescope applied sharper band-pass filters and thus can mitigate the problem to a certain extent. In contrast, the ring focal design for broadband signal reception used for the TWIN telescopes is considerably more sensitive to RFI effects. S-band results are indeed expected to be less precise compared to X-band data, because group delay precision is a function of the signal bandwidth which is only approximately 200 MHz in S-band for RTW in our legacy mode, whereas it is 720 MHz in X-band. However, the discrepancy between S- and X-band results is drastic and underlines significant RFI problems. In many cases, the outcome is simply not usable in geodetic VLBI.

In order to demonstrate the suitability of the TTW1 telescope on a long baseline for the use of both, X- and S-band data, we analyzed one 24-h session (20 August 2014) including six telescopes all over Europe. In this analysis, we investigated in particular the influence of the ionosphere correction term to the group delay observations as a simple linear combination of two group delay observables (X- and S-band). In this context, we generated a solution excluding the observations of the RTW-TTW1 baseline in Wettzell to determine its baseline length only with observations from long baselines throughout Europe. Comparing this solution (including X- and S-band data) to the results obtained by the Whisp sessions using the short RTW-TTW1 baseline (using X-band data only) leads to an evidence of the suitability of the S-band data. The results of this study are shown in Section 4.3.3.

#### 4.3.2. X-Band Results

**Figure 15:**
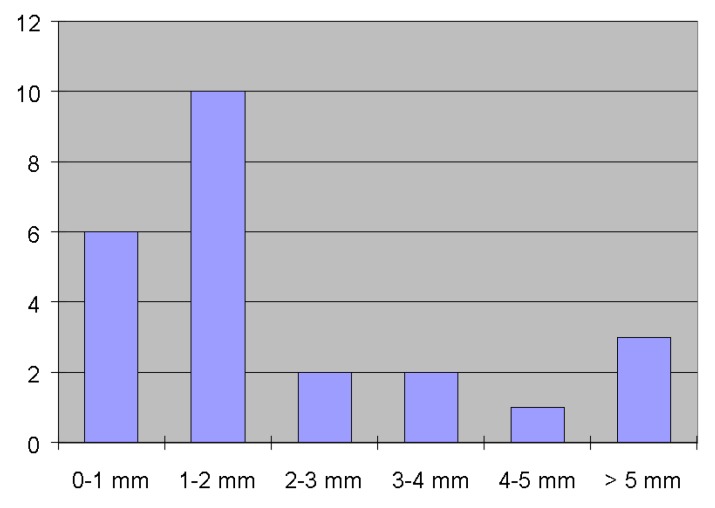
Histogram of the 3D distance discrepancies as found from X-band results in Table 6. The number of occurrences (24 experiments with the initial S/X-band receiver) is given in y-direction, the histogram bin in x-direction.

In contrast to the results retrieved from S-band group delays, the results derived from X-band group delays are very encouraging and satisfactory, regarding the use of both the DBBC (Table 6) as well as the ADS3000+ backend (Table 7). Figure 15 shows a histogram of the 3D differences (reference distance from local survey) and is related to the 24 experiments listed in Table 4. The deviation is less than or equal to 2 mm in 16 experiments. The three experiments covering 24 h each, Europe (20 August 2014), Whisp1 (27 August 2014) and Whisp2 (23 October 2014) are off by +1.1, −1.8 and −0.8 mm respectively.

Regarding the data sampled with the ADS3000+ (Table 7), we can see that one Intensive experiment (22 September 2014) with 20 observations (19 in use, one was rejected) shows a difference of around −9.1 mm, whereas the corresponding experiment sampled with the DBBC deviates by 4.4 mm which is also higher than the majority, but only half of the deviation of the ADS3000+.

The number of rejected scans is moderate in most cases. Only eight scans out of 661 group delays were rejected in the Whisp experiment adjustment, and only two scans in the Europe experiment. The percentage is higher, although not yet critical, in the second Whisp experiment (23 October 2014) with 52 rejections (5%). The first Intensive experiment analyzed resulted in six rejections (24%) with the first five observations clearly being gross errors (residuals between 3 and 22 ns corresponding to 0.95 to 6.8 m), probably due to a failure during VLBI correlation. The situation is different for the Intensive carried out on 6 October 2014 with four rejections (17%): In this case, the observations identified as outliers had residuals around 27 to 51 ps (8 to 15 mm), which is higher than for the accepted data (only up to 14 ps), but still at a level of magnitude not indicating gross failures in the correlation or sampling process.

As far as the two experiments conducted using the new tri-band receiver (23 February and 2 March 2015) are concerned, we find a slightly higher deviation from the nominal 3D distance compared to the results obtained using the initial S/X-band receiver. Although these two initial results are not enough to draw clear conclusions regarding the performance of the tri-band system, separate technical investigations revealed some minor shortcomings which have to be mitigated: An apparent problem in the third Nyquist zone (1024–1536 MHz) visible as significant DC-level in the cross-power spectrum of the correlator report could be identified as a inappropriately adjusted power level at the input of the digital down-converter (DBBC-2).

The standard deviation of unit weight *a posteriori* agrees reasonably well with that *a priori*. Looking at Table 6 (DBBC backend), all standard deviations *a posteriori* are slightly higher (10 to 35 ps) than *a priori* (7 to 30 ps), but at a similar level. Regarding the ADS3000+ backend (Table 7), we can see even slightly smaller or equal standard deviations *a posteriori* in three cases out of the six experiments performed.

##### Quick Glance at the Whisp1 Experiment (27 August 2014)

**Figure 16:**
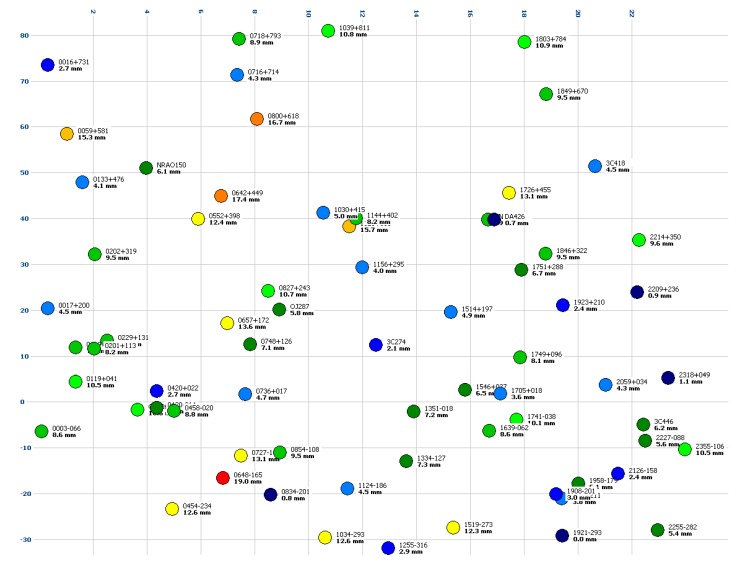
Color-coded maximum residual per radio source for the “Whisp1” experiment (27 August 2014) in X-band using the DBBC backend. The coordinate system is right ascension α (*x*-direction, in hours) *versus* declination δ (*y*-direction, in degrees). The radio source name is printed with the max. residual in units of millimeters below. Minimum and small residuals in blue and green, large and maximum residuals in orange and red.

**Figure 17:**
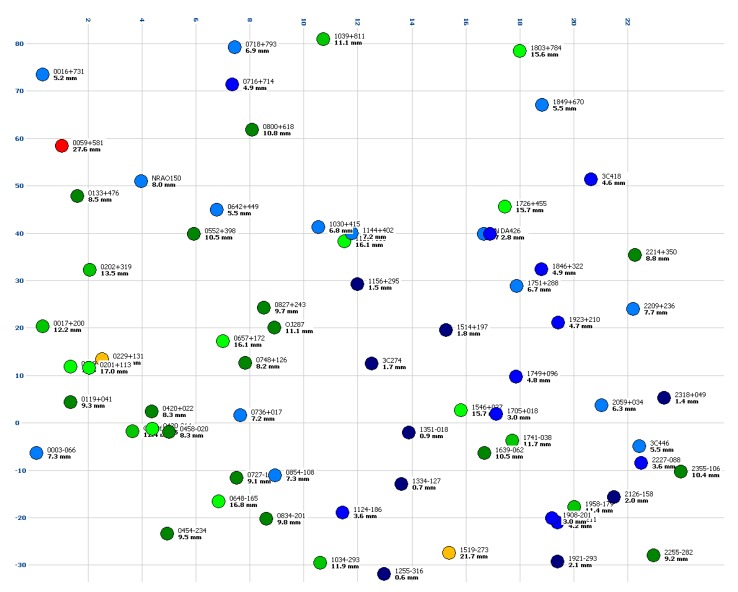
Color-coded maximum residual per radio source for the “Whisp1” experiment (27 August 2014) in X-band using the ADS3000+ baseband converter. The coordinate system is right ascension α (*x*-direction, in hours) *versus* declination δ (*y*-direction, in degrees). The radio source name is printed with the max. residual in units of millimeters below. Minimum and small residuals in blue and green, large and maximum residuals in orange and red.

Both the DBBC and the ADS3000+ were integrated for the first Whisp experiment observed by the TTW1 telescope. The receiving system is, of course, identical, but the backend and recording was implemented independently. Figure 16 (DBBC) and Figure 17 (ADS3000+) show a plot of the maximum residuals in a coordinate system featuring right ascension α (x-direction) and declination δ (y-direction). Note that one and the same radio source was observed multiple times. In this diagram, however, the source can only be plotted at its unique α,δ-coordinates which do not change in time. As a consequence, only the maximum (unsigned) residual is plotted.

When comparing the two diagrams we do not see any systematic patterns. The results obtained with the DBBC and the ADS3000+ backend appear to be stochastically independent. We consider this to be an indication that no dominant systematic error effects are introduced via the receiving chain, because such effects should yield similar patterns in the residual plot. In our case, the distribution appears to be random, although this topic might still require a more detailed investigation. Note that the phase calibration unit of TTW1 was disabled for the local experiments as mentioned in Section 4.2.

#### 4.3.3. Alternative VLBI Data Analysis Using Calc/Solve

In addition to the in-house software used at the Geodetic Observatory Wettzell, the VLBI group at the Institute of Geodesy and Geoinformation of the University of Bonn performed a comparative data analysis using the Calc/Solve software package and the corresponding solution setup described in Section 4.1.2.

We selected experiments with 24-h observation periods for our comparisons. Up to now, only three experiments have been observed which cover the full 24-h period (Europe-130 on 20 August 2014, Whisp1 on 27 August 2014 and Whisp2 on 23 October 2014). These data are processed for the estimation of station coordinates on a session-by-session basis:

*Short baseline analysis*: Only X-band observations on the short 123 m baseline between RTW and TTW1 are included in the first solution setup, from which the relative telescope coordinates are estimated. The baseline lengths are deduced from the coordinate results and listed in Table 8. The resulting baseline lengths RTW-TTW1 differ only in the range of a few millimeters for the three experiments. We compare the individual results to the mean of the two Whisp sessions here. One reason to exclude the Europe session from this mean is that the number of observations is significantly smaller than for the two Whisp sessions. The second reason is that we want to establish reference results which are completely independent of the Europe session because we use these session’s data in Section 4.3.1 for validating the S-band data.

**Table 8:** Results obtained from X-band group delays using the DBBC backend and the VLBI analysis software package Calc/Solve.

Date	Type	No. Obs.	3D Distance and Std. Deviation		Mean Length and Std. Deviation		Residuals
			(mm)	(mm)	(mm)	(mm)	(mm)
20 August 2014	E	219	123,307.4	15.1	**123,307.5**	12.0	−0.16
27 August 2014	W	652	**123,307.1**	14.8			−0.39
23 October 2014	W	1000	**123,307.9**	15.8			0.39

The resulting reference baseline length from VLBI data analysis differs only 0.5 mm from the value obtained by terrestrial measurements. Thus, the determination of the small baseline length RTW-TTW1 was accomplished rather successfully.

*Long baseline analysis*: In addition to the baseline vector determination, the quality of the S-band data is further investigated in the following. As outlined and discussed in Section 3.2 as well as Section 4.3.1, S-band data are of inferior quality due to remaining RFI problems in the single S-band data. In order to demonstrate the suitability of the S-band data, in particular for long baseline processing using either the TTW1 or the RTW telescope and a partner telescope, we focus on one 24-h session including both, small distances at Wettzell as well as long baseline lengths throughout Europe. For this purpose, the data from the Europe-130 experiment (20 August 2014), which consists of six VLBI stations (Madrid, Spain; Metsahovi, Finland; Ny Alesund, Norway; Zelenchukskaya, Russia; TTW1 and RTW in Wettzell, Germany) are used.

In this test analysis, we deliberately excluded the observations of the short 123 m baseline between RTW and TTW1 and determined the vector between these telescopes only with observations on the long trans-European baselines. Here, the X-band group delay observables are corrected for ionospheric refraction through the co-linear S-band observations.

Analysis yields a Wettzell local vector length of 123.3089 m which agrees very well with the mean of the Whisp sessions (123.3075 m) as well as the reference distance from local surveying (123.3070 m) considering the fact that the results were deduced from completely independent session types. It should be mentioned that the shortest baseline is the one to Madrid with a length of about 1650 km.

In conclusion, regarding the short TTW1-RTW baseline, the S-band data are indeed affected by RFI problems as mentioned in Section 4.3.1. However, as indicated in Section 3.2, the S-band can be successfully used for ionosphere calibration on the long trans-European baselines leading to satisfactory results.

## 5. Conclusions and Outlook

TTW1, the first of the two new TWIN VLBI radio telescopes at the Geodetic Observatory Wettzell, Germany, is a fast-moving antenna system that was developed according to VGOS standards in major parts. The main difference between TTW1 and the second TTW2 telescope is the use of a tri-band feed horn with reception capabilities in S-, X- and Ka-band, whereas a 2–14 GHz broadband feed will be used for TTW2. The commissioning phase for TTW1 started in June 2014. During this period, the antenna accompanied selected standard experiments in parallel (“tag-along mode”) with the existing 20 m RTW radio telescope. Most of these experiments where so-called “Intensives” with a duration of one hour each. In addition, three extra experiments with a duration of 24 h where conducted.

Correlation results indicate a very satisfactory quality of the X-band data, but radio frequency interference problems in the S-band. However, correlation on long baselines causes local RFI to decorrelate. This principle yielded a successful demonstration regarding the initial processing results. Local positioning performance using X-band data shows deviations of usually less than 2 mm (3d distance between TTW1 and RTW) in the majority of the experiments.

Despite of the fact that long baseline correlation of S-band data yields useful group delays, internal investigations reveal that TTW1 is more affected by RFI problems due to its broad band design (ring focus antenna) compared to the traditional RTW system. Consequently, one of the action items is to investigate technical solutions for more efficient RFI resistance carefully, using sharp band-pass filters for instance, and to implement suitable solutions accordingly. Moreover, the initial results presented in this paper are based on group delays obtained in RTW-compatible mode. More and extensive tests exploiting the advanced features of this VGOS antenna are pending, in particular data sampling in dual-polarization mode, usage of broader frequency bands (1 GHz) in correlation as well as the analysis of Ka-band data. The latter two aspects can be dealt with as soon as the TTW2 or another suitable partner telescope is becoming available.
